# A Mathematical Model of Metformin Action on COVID-19 Risk Infection in Cardiovascular Diabetic Patients Studied by FTIR Spectroscopy

**DOI:** 10.3390/ijms26136332

**Published:** 2025-06-30

**Authors:** Evangelos Mylonas, Christina Mamareli, Michael Filippakis, Ioannis Mamarelis, Jane Anastassopoulou, Theophile Theophanides

**Affiliations:** 1 Department of Digital Systems, University of Piraeus, 80 M. Karaoli & A. Dimitriou, 18534 Piraeus, Greece; evanmylonas7@gmail.com (E.M.); mfilip@unipi.gr (M.F.); 2 Athens Institute for Education and Research, 9 Chalckokondili Str., 10677 Athens, Greece; christinamma32@yahoo.gr (C.M.); theo.theophanides@gmail.com (T.T.); 3 Cardiology Department, 401 Military General Hospital, Mesogeion Av. 138 & Katechaki Georgiou Str., 11527 Athens, Greece; imamarelis@gmail.com

**Keywords:** COVID-19, diabetes, metformin, insulin, thiazolidinediones, FTIR spectroscopy, infected and recovered, mathematical model

## Abstract

Several studies have revealed that patients with type 2 diabetes (T2D) infected with COVID-19 who were medicated with metformin showed higher recovery rates than those administered other antidiabetic drugs. To determine the mechanism of action of antidiabetic drugs against COVID-19, we developed a mathematical model that was based on the number of infected and recovered T2D patients. Moreover, the “diagnostic frequencies” of the infected T2D patients, determined using Fourier-Transform Infrared (FTIR) spectroscopy, were very helpful. In particular, the band at 1775 cm^−1^, attributed to IgG antibodies, could be used as a “diagnostic frequency” for COVID-19 infection. The increased intensity of the band of *v*C-O-C sugar moieties suggests an increased number of OH chemical groups that enhance the binding sites of SARS-CoV-2 spike protein for entering host cells. The changes were more pronounced in patients medicated with thiazolidinediones than those using insulin and metformin. Both FTIR spectra and the developed mathematical model confirmed that patients using thiazolidinediones showed a higher risk of COVID-19 infection and mortality. The data support the hypothesis that the NH chemical groups of metformin molecules interact directly through the SARS-CoV-2 spike protein, preventing the entry of COVID-19 into the host membrane cells. Indirectly, metformin inhibits the host binding sites for COVID-19 entry by lowering AGE production.

## 1. Introduction

Diabetes is a lifelong metabolic disease, with an increasing tendency worldwide. It has been estimated that it affects about 6.1% of the Greek population [1]. Diabetes is characterized by high blood serum glucose that disturbs carbohydrate and lipid metabolism and is associated with several secondary implications, such as heart attack and stroke, leading to high global mortality rates. COVID-19 has infected many people, while epidemiological, clinical, and laboratory investigations have revealed that patients with cardiac comorbidities have an increased risk of mortality. Numerous studies have shown that, among all risk factors, diabetes was the leading factor for longer hospitalization and increased mortality post-infection with the zoonotic pathogen COVID-19. However, metformin (MET), an antidiabetic medication used in the treatment of type 2 diabetes (T2D) patients, showed the lowest mortality rate among all the available antidiabetic agents [2,3,4,5].

Since the COVID-19 pandemic first emerged worldwide, researchers have focused on evaluating the epidemic incidence rate of the virus, as well as analyzing and predicting the parameters that affect its spread in a region. Several mathematical models have been developed to estimate the transmission rates among symptomatic and non-symptomatic infected people [6,7,8,9,10]. Despite the existing mathematical models in the literature, we aimed to develop a mathematical simulation model that assesses the risk of morbidity and mortality from COVID-19 in T2D patients based on their antidiabetic medication [10,11]. To determine the molecular mechanism of SARS-Cov-2 action in relation to antidiabetic medication, Fourier-Transform Infrared (FTIR) spectral data were also used to detect the characteristic “diagnostic bands” used to study the influence of antidiabetic medication on COVID-19 infection [10,11,12]. FTIR spectroscopy highlights the “fingerprint region” at a molecular level of the studied tissues. The infrared frequencies of molecular absorption bands depend on the vibrational energy of the chemical bonds of functional chemical groups and the surrounding environment of the molecules. In reality, the changes caused by the disease affect the dipole moment of the chemical bond of functional groups, OH, NH, etc. Thus, any change in the intensity or frequency shifts in the absorption bands arises from the disease, which affects the vibrational frequencies [1,13,14].

The focus of the present research is to develop a mathematical simulation model to approximate the risk of morbidity and mortality from COVID-19 in diabetic patients based on their antidiabetic medication. FTIR spectroscopy provided significant benefits in determining the molecular mechanism of SARS-CoV-2 action and the influence of antidiabetic medication on T2D patients. FTIR spectroscopy is an easy-to-use, fast, non-destructive, and sensitive physicochemical method, where the spectrum comprises the patient’s fingerprint. In recent years, FTIR spectroscopy has also been applied as a diagnostic tool in medicine and biology [15].

## 2. Results

### 2.1. FTIR Spectra

**Figure 1:**
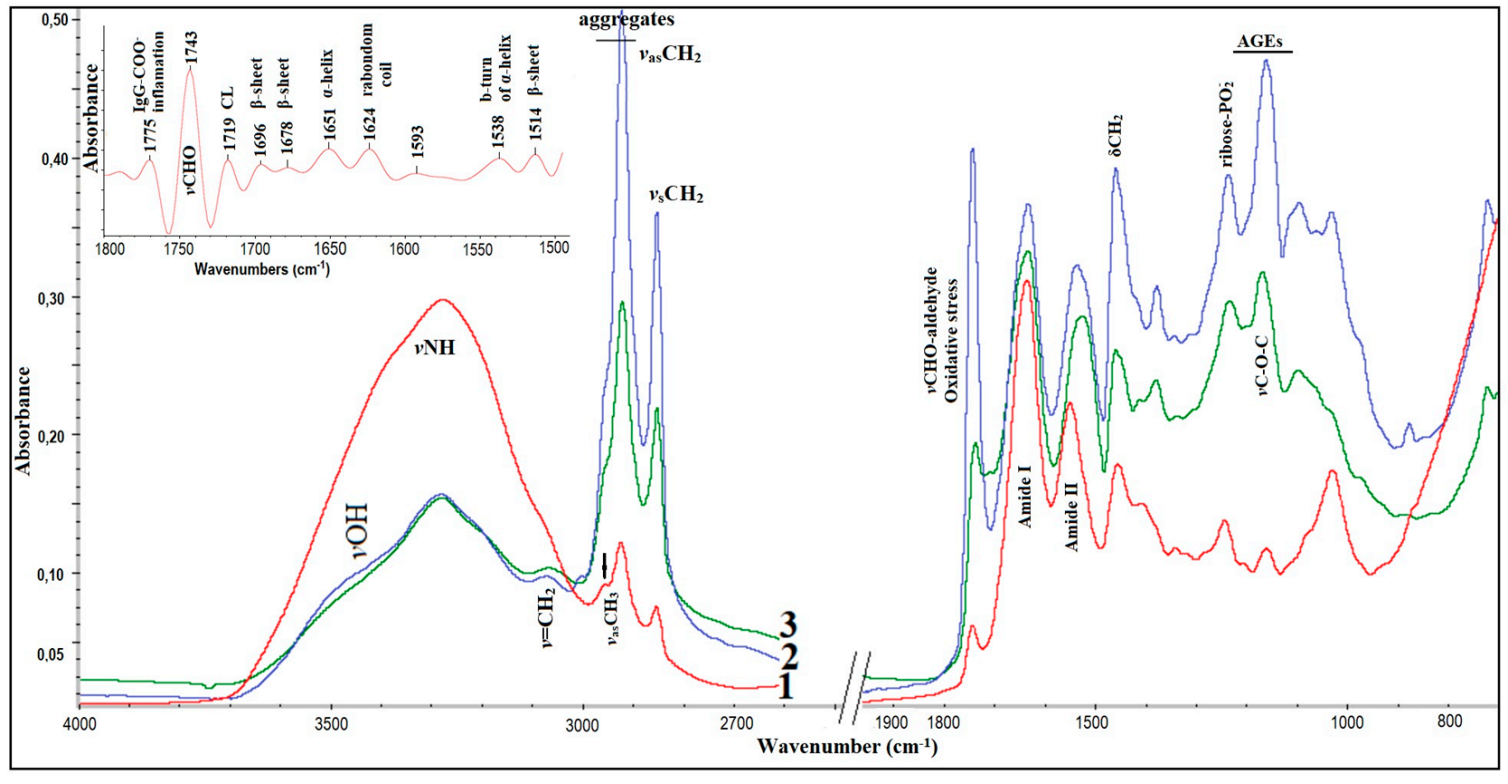
Representative FTIR spectra from T2D patients correlated with antidiabetic medication: **1** metformin (MET), **2** thiazolidinedione (TZD), and **3** insulin in the spectral region 4000–800 cm^−1^. The inserted FTIR spectrum shows the deconvoluted FTIR spectra of T2D patients medicated with TZD (spectrum 3) in the 1800–1500 cm^−1^ region. The spectra were recorded at room temperature with an ATR (attenuated total reflection) apparatus in the spectral regions of 4000–2600 cm^−1^ and 1800–800 cm^−1^.

The characteristic FTIR spectra of coronary arteries obtained from the T2D patients medicated with MET (1), insulin (2), and TZD (3) are presented in Figure 1. The spectra were recorded in the 4000–2600 cm^−1^ and 1800–800 cm^−1^ regions because we noted that the spectra were affected by diseases in these regions [1,8,9,11,12]. A comparison between them showed noticeable differences in the absorption band shape, intensity, shifts, and the appearance of new bands related to the disease and affected by the medication.

The high-intensity decrease in the *v*NH stretching vibration band at about 3300 cm^−1^ in both spectra of TZD- and insulin-medicated patients indicates reduced protein NH groups and α-helix native secondary structure changes caused by disease, mostly to β-sheet configuration [1]. This suggestion is also supported by the appearance of a shoulder peak at about 3250 cm^−1^, indicating β-sheet conformational structure formation [1,16]; it is also supported by the intensity increase in the antisymmetric and *v*_as_CH_2_ symmetric stretching vibration bands in the 3000–2850 cm^−1^ spectral region due to increasing lipophilic environment of membranes caused by the disease, while the *v*_as_CH_3_ and *v*_s_CH_3_ stretching vibration bands almost disappear [1], compared with MET-medicated patients. In healthy tissues, the antisymmetric and symmetric vibration bands of methyl (*v*_as,_*v*_s_ CH_3_) and methylene (*v*_as,_*v*_s_CH_2_) groups are present at very low intensities [1]. The appearance of such in the TZD-medicated patient spectra of the new “diagnostic band” near 1775 cm^−1^ (as shown in the insert spectrum in Figure 1), attributed to immunoglobulin-G(IgG) glycosylated carbonyl groups, is crucial, as it predicts COVID-19-induced protein glycosylation and inflammation [17,18,19]. This new band could be used to identify the cardiolipin antibodies caused by COVID-19 [20]. The absorption band at 1719 cm^−1^ results from the cardiolipin molecules located between the two layers of the cell membrane [1,20]. Moreover, the band at 1719 cm^−1^ shows that an acidic environment (acidosis) was developed during the disease [1]. The last two bands could also be used as “diagnostic and signature bands” of COVID-19 etiology, which causes pathological problems characterized by enhanced oxidative stress, such as pro-inflammatory and inflammatory effects, followed by glycosylation [17,18,19,20]. The “diagnostic band” at 1743 cm^−1^, assigned to the aldehyde stretching (*v*CHO) absorption mode, was increased in the spectra of TZD- and insulin-medicated patients compared with that of MET-medicated patients, confirming that COVID-19 heightens oxidative stress and aldehyde formation effects [10,14].

The amide I (1650 cm^−1^) and II (1645 cm^−1^) absorption bands of native α-helix conformation proteins overlapped with the new weak bands 1691 cm^−1^, 1626 cm^−1^, and 1540 cm^−1^, originating from protein parallel and antiparallel β-sheet formation due to protein glycosylation from diabetes- and COVID-19-induced oxidative stress [1,8]. It was observed that COVID-19 altered β-sheet conformer production. Band intensities were less pronounced in the spectra of MET-medicated patients than TZD-medicated patients. β-sheet conformers originated from interactions between protein strands held together with hydrogen bonds (H-bonds) and enhanced by the lipophilic environment [16]. The observed increase in antisymmetric and symmetric absorption bands of methylene groups in the 3000–2850 cm^−1^ region supports this hypothesis.

The most suitable spectral region for studying the effects of diabetes and COVID-19 on patients is located between 1250 and 900 cm^−1^. In this spectral region, *v*PO_2_^−^ absorption bands sensitive to disease progression are determined: *v*C-O-C of glycans and *v*C-O-P of phospho-ribose groups from RNA and DNA. The spectral band shape and position are related to advanced glycation end products (AGEs) after protein glycosylation and phosphorylation. Furthermore, deconvoluting the 1300–900 cm^−1^ spectral region (spectra not shown) revealed shape and intensity changes. The new bands observed in the 1200–900 cm^−1^ region in the TZD-medicated patient spectra suggest the production of several different glycosylated products, yielding immune responses upon viral infection. The subunit COVID-19 spike glycoprotein consists of C- and N-terminal domains, which play important roles in viral entry by binding to the -OH and -NH functional groups of sugars and proteins, activating various oxidative stress and inflammation pathways.

### 2.2. Mathematical Model

The classical susceptible-infected-recovered (SIR) model provides an easy way to understand the influence of the disease on the population, using the fundamental Equation (1) [21]:

(1) $$S^{\prime }\left(t\right)=\frac{-b}{N}R\left(t\right)I\left(t\right)$$

where S(t), I(t), and R(t) are the susceptible, infected, and recovered patients, respectively, as a function of time.

However, the classical SIR model does not involve parameters that approximate the more complicated problems caused by existing human diseases, which affect disease severity, such as diabetes, cholesterol levels, etc. For multivariant biological phenomena, we suggest fractional-order calculus (FOC) as an ideal mathematical system for describing the COVID-19 dynamics and explaining the aspects regarding the medications of infected T2D patients. The FOC model contains more degrees of freedom, which are associated with non-local phenomena, and we suggest that the model could describe the qualitative properties of the solutions for the medication effect on morbidity and mortality for T2D patients infected with SARS-CoV-2. In this case, Equation (2) yields the FOC for including the risk factor of SARS-CoV-2 infection in association with diabetes and drug pharmacodynamics [22].

(2) DaxγCft=1Γn−γ∫axfnst−sγ+1−nds

where f(t) is a continuous function of n + 1 times differentiable in the interval α,χ, which are the upper and lower limits of the integral evolution of COVID-19, and γ represents the fractional order with limits γ ϵ (0,1). Theoretically, the fractional order of γ = 0 and γ = 1 denote the T2D patients who did not receive any medication and the general population, respectively. The parameter Γ signifies the Gamma Euler function, which allows us to obtain the arithmetical values of COVID-19 affinity in relation to medication, which is given by Equation (3):

(3) $$\Gamma \left(n\right)=\left(n-1\right)!\;\mathrm{di}$$

where n is a positive integer, and ! is the factorial of a number given by Equation (4):

(4) $$\left(n-1\right)!=1\times 2\times \ldots \ldots .\times n-1$$

In this case, the extended proposed model is described by the derivative Equation (5):

(5) DaxγCSt=βStIt

For the infected diabetic patients, the derivative equation is given by Equation (6):

(6) DaxγCIt=βStIt−It

Parameters β and θ measure the COVID-19 infection rate with respect to time and the possibility of COVID-19 recovery, respectively. The recovered diabetic patients are calculated from Equation (7).

(7) DaxγCRt=1−θβSt+ It

This parameter includes the entire history of COVID-19 and can be considered a risk factor indicator. To correlate the antidiabetic drug activity with COVID-19 risk factors, the system of Equations (5)–(7) was numerically applied to T2D patients in each medication category.

To numerically approximate the solutions of Equations (5)–(7), we used a modified form of the Predictor–Corrector Method (PECE). It is worth noting that the Predictor–Corrector Method was initially used by Choi et al. [23]. The PECE was applied to Caputo fractional differential equations in the following form [24]:

(8) DaxγCyt=ft,yt

The PECE is primarily based on the trapezoidal quadrature formula. At first, we assumed that our general function y(t) was continuous on an interval of the form [0,T]. Choi et al. sequentially step t = nk, a positive integer, and k denotes the size of our step. We used the following integral form based on the fundamental theorem of calculus via induction:

(9) $$y\left(t_{n+1}\right)=y\left(t_{n}\right)+\int_{t_{n}}^{t_{n+1}}f\left(s,y\left(s\right)\right)ds$$

By using the trapezoidal quadrature formula to define our integral term, we reach the following equation:

(10) $$\int_{a}^{b}f\left(s\right)ds=\frac{f\left(a\right)+f\left(b\right)}{2}$$

Equation (9) leads to the following form (11):

(11) $$y\left(t_{n+1}\right)=y\left(t_{n}\right)+\frac{k}{2}\left(f\left(t_{n}\right),y\left(t_{n}\right)\right)+f\left(t_{n+1},y\left(t_{\left(n+1\right)}\right)\right)$$

Using the implicit one-step E–Bashford–Moulton method, replacing $y\left(t_{n}\right),y\left(t_{n+1}\right)$, we recursively obtain Equation (12):

(12) $$y_{n+1}=y_{n}+\frac{k}{2}\left(f\left(t_{n},y_{n}\right)+f\left(t_{n+1},y_{n+1}\right)\right)$$

Although it seems impossible to solve the equation, since the function f(t,y(t)) is non-linear, we can modify the initial conditions of the application. At this point, we similarly introduced a predictor value, replacing the trapezoidal quadrature formula with the rectangular rule that transforms it into the well-known Euler Method:

(13) $$y_{n+1}^{p}=y_{n}+kf\left(t_{n},y_{n}\right)$$

Equation (12) is modified to the final numerical Equation (14):

(14) $$y_{n+1}=y_{n}+\frac{k}{2}\left(f\left(t_{n,}y_{n}\right)+f\left(t_{n+1},y_{n+1}^{p}\right)\right)$$

**Figure 2:**
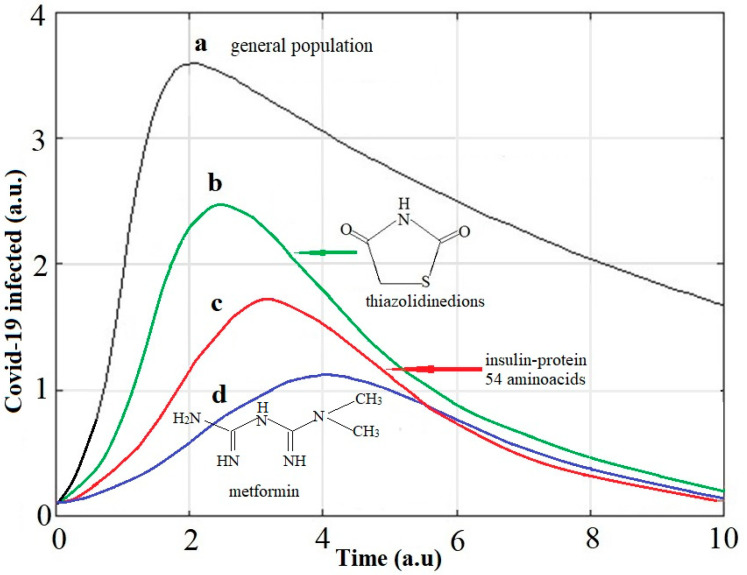
Relationship between type 2 diabetes (T2D) patients infected by COVID-19 and risk of mortality associated with antidiabetic medication. The curves correspond to the general population (**a**); thiazolidinediones, TZD (**b**); insulin (**c**); and metformin, MET (**d**).

Under this methodology, patients were organized into four categories: the general population and TZD-, insulin-, and metformin-medicated T2D patients [25,26]. For the solutions of the FC equations studied, the parameter γ receives the maximum values of γ = 1, 0.9, 0.5, and 0.3 for the general population, T2D patients medicated with TZD, insulin, and metformin, respectively. Evidently, γ cannot receive the value 0, since there is no T2D patient without any antidiabetic medication. Figure 2 illustrates the effects of the three antidiabetic drugs—TZD, insulin, and MET—on T2D patients compared with the general population affected by COVID-19.

The mathematical model shows that the general population (Figure 2a) is at a higher risk of mortality compared to all T2D patients. To interpret this result, we consider that the general population comprises individuals of all ages who may have diabetes or other diseases but have not been diagnosed yet [25]. There is also strong evidence of the protective role of MET (Figure 2d) against COVID-19 compared to insulin and TZD. These results are consistent with the reported clinical data that T2D patients using MET were not hospitalized or did not require intensive care unit admission [3,27,28]. The protection role of MET is also supported by the intensity-reduced FTIR spectra “marker band” at 1743 cm^−1^. Retrospective studies demonstrated that mono-insulin therapy in T2D patients had a much higher rate of mortality [29]. However, our model showed that insulin-dependent T2D patients are at higher risk than those treated with TZD. This discrepancy in our study could be explained by the suggestion that T2D patients treated with insulin are medicated with at least one more antidiabetic drug. In general, the mortality of T2D patients follows the medication therapy in the order TZD > insulin > MET, as validated by GNPHO and other clinical data (see Table 1) [25,26].

## 3. Discussion

The FTIR spectra of patients provided important information about the effect of COVID-19 on diabetic patients using different antidiabetic agents. The FTIR spectroscopic “diagnostic bands” predicted that COVID-19 enhanced the induced oxidative stress, leading to increased IgG, protein-folding dysregulation, and non-reversed AGEs. It is documented that heterogenous AGEs from non-enzymatic redox reactions propagate oxidative stress and promote inflammation, triggering the signaling pathways of diabetes complications [30,31]. The reduced-intensity absorption bands at 3300 cm^−1^, in turn, reduced the number of -NH groups. We suggest that -NH groups of aminoglycans and proteins’ amino acids are linked to -OH sugars of spike protein with H-bonds. This hypothesis is supported by the observations of other investigators who used NMR (nuclear magnetic resonance) spectroscopy to characterize the preference of COVID-19 spike binding to host via N-link glycans with lectins [32]. They also found that COVID-19 spike glycoproteins bind to nitrogen (N) atoms of glycans and form intermolecular hydrogen bonds with their -OH groups. Since AGEs contain an excess number of functional chemical -OH and -NH groups, they can enhance the targeting sites of the host tissue for spike glycoproteins, which attack via hydrogen bonding, promoting viral entry. These observations align with the FTIR spectral bands, which show increased AGEs in insulin- and TZD-medicated T2D patients. Considering that both COVID-19 spike S-glycoprotein and AGEs contain -OH and -NH groups, they can interact through hydrogen bonds in a donor–acceptor model, which facilitates viral entry to host cells. Furthermore, the binding site of the COVID-19 spike consisted of β-sheet subdomains, a structure like those caused by diabetes and enhanced by COVID-19. The spectral data strongly indicated that the 1250–900 cm^−1^ spectral region could be used as a “biomarker region” for COVID-19 severity diagnosis. For TZD- and insulin-medicated patients, the FTIR spectra also showed considerable differences in the 1800–900 cm^−1^ spectral region. The increasing absorption band at 1734 cm^−1^, corresponding to phospholipid and cholesteric esters, strongly supported the suggestion that lipid hydroperoxidation most likely occurred due to oxidative stress caused by COVID-19 in TZD- and insulin-medicated patients. These bands were less pronounced in MET-medicated patients (Figure 1). Overall, the observed changes in FTIR “diagnostic bands” and the developed mathematical simulation model reflect the influence of antidiabetic medication on the COVID-19 binding affinity to diabetic patients.

**Figure 3:**
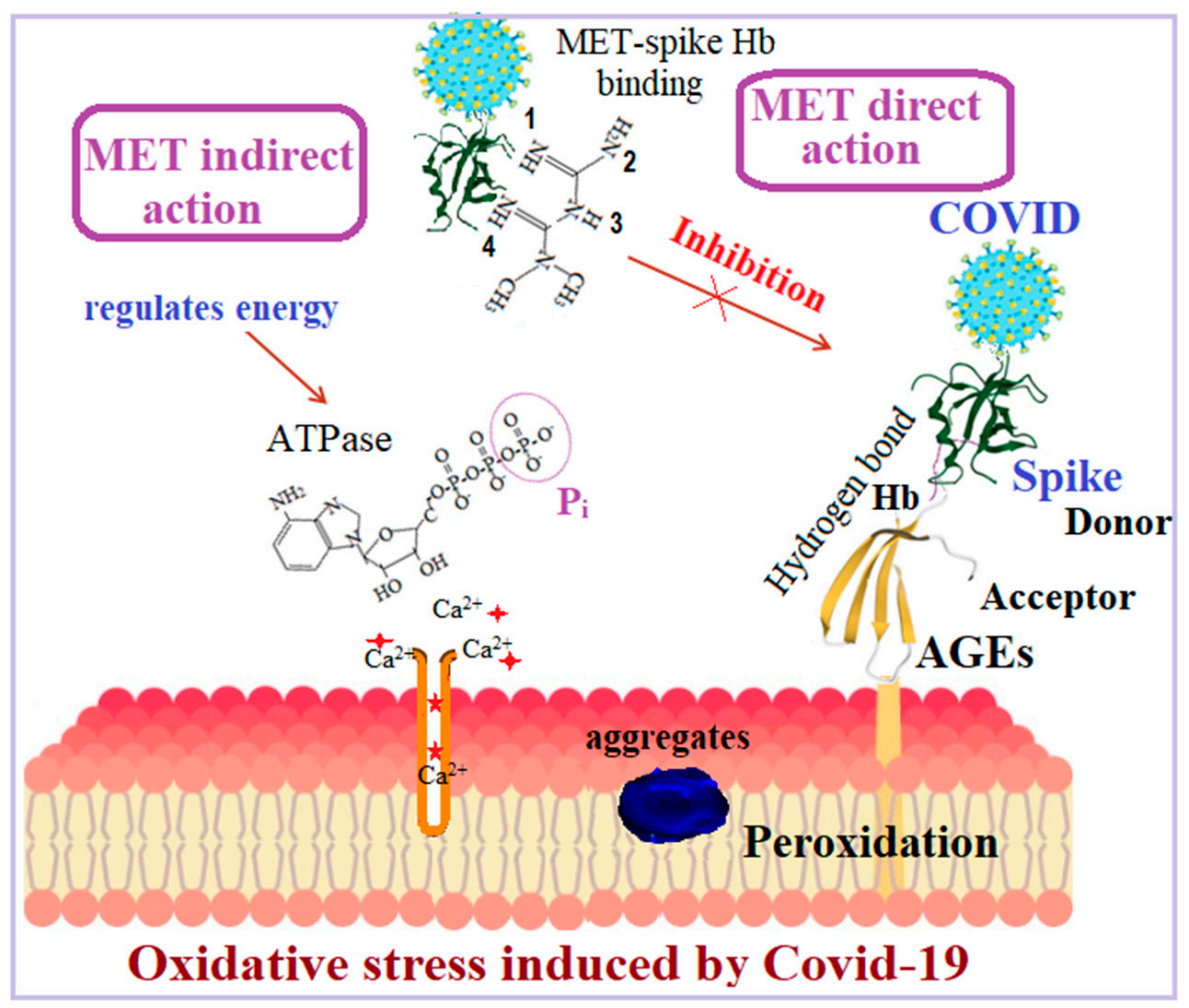
Schematic presentation of metformin’s dual action on COVID-19. Direct action: Each of the four nitrogen atoms of metformin could act intermolecularly via hydrogen bonding with -OH groups of COVID-19 spike glycoprotein. Indirect action: MET regulates the ATPase and inhibits calcium cation (Ca^2+^) release, AGE production, and the redaction of the binding sites of the host (cells).

Based on both the FTIR data and the mathematical model, it was suggested that the key severity of COVID-19 effects on T2D patients depended on the pharmacokinetic properties of antidiabetic drugs. Thus, TZD acts on cytochrome P450, which supports electron transfer reactions, altering the redox potential of the cells, triggering other metabolic disorders. Trends from past studies show that TZD-induced weight gain and edema aggravate heart failure [33,34]. These results do not support the use of TZD in patients suffering from COVID-19. More clinical trials are needed to optimize the risk-to-benefit ratio of using this drug in patients with COVID-19. Many studies have reported that patients using insulin have poor prognoses due to increased renal angiotensin-converting enzyme 2 (ACE2) expression [28,35]. Using various physicochemical methods, many researchers have suggested that SARS-CoV-2 spike protein interacts with host cells via H-bonds [32,33,36]. The FTIR spectral “diagnostic bands” support the hypothesis that MET protects T2D patients and reacts directly with SARS-CoV-2 spike protein via H-bonds, as schematically presented in Figure 3.

**Scheme 1:**
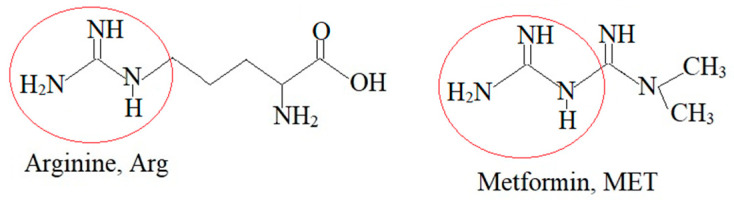
Chemical molecular structures of Arginine and Metformin. The circles underline a similar part of the two molecules, which may indicate a similar attachment to COVID-19.

As shown in Figure 3, the MET molecule contains four nitrogen atoms, which bind directly to SARS-CoV-2 spike glycoproteins to form intermolecular H-bonds with N1, N2, N3, and N4 atoms or 1N-2N, 1N-4N, and 2N-3N couples. The reactions between MET and SARS-CoV-2 spike protein binding to cells were established as donor–acceptor reactions. It has been observed that the S-spike glycoproteins of both SARS-V and SARS-CoV-2 (COVID-19) interact with the ACE2 (angiotensin-converting enzyme 2) receptor in the cholesterol-rich region on the host cell membrane, leading to entry into the target cells [35]. Jafary et al. established that the preferential binding of SARS-CoV-2 spike glycoprotein is the N-site of Arginine (Arg) and Tyrosine (Tyr) amino acids using ACE2 in their in silico investigation [36]. MET contains an identical part with Arg in its molecular structure, as shown in Scheme 1 (circles), suggesting that MET could bind directly with SARS-CoV-2 spike glycoprotein in a similar way.

Furthermore, the two N=C double bonds of MET molecules are strong scavengers of hydroxyl radicals, produced during oxidative stress, and may inhibit AGE formation and inflammatory responses [36,37]. Additionally, MET regulates AMP kinase (adenosine monophosphate), inhibiting AGE accumulation and minimizing inflammatory responses [38]. These pathways lead to ATP (adenosine triphosphate) reproduction and Ca^2+^ cation homeostasis.

The FTIR spectra of the ex vivo biopsies provided important information about the effect of COVID-19 on diabetic patients using different antidiabetic agents. The FTIR spectroscopic “diagnostic bands” predicted that COVID-19 enhanced the induced oxidative stress, leading to increased IgG, protein-folding dysregulation, and non-reversed AGEs. The literature documents that heterogeneous AGEs, from non-enzymatic redox-propagated reactions due to oxidative stress, promote inflammation and trigger the signaling pathway of diabetes complications. COVID-19 also promotes glycolytic reactions and amplifies the number of AGEs in patients, leading to other pathological pathways [25]. Protein glycosylation produced an excess of terminal -OH and -NH groups, which enhanced the binding sites of the host cells in tissues for spike glycoproteins to attack via hydrogen bonding, promoting viral entry. These observations align with literature data concerning the spike molecular structure [29,30,31,32,33]. Considering both SARS-CoV-2 spike glycoprotein and AGEs contain -OH and -NH groups, they can interact via H-bonds in a donor–acceptor model, which facilitates the virus’s entry into host cells. Furthermore, the binding site of SARS-CoV-2 spike consists of β-sheet subdomains, a similar structure to that formed in all diabetic patients [1] and enhanced by the virus infection. The spectral data strongly indicated that the spectral region 1250–900 cm^−1^ could be used as a “biomarker region” for COVID-19 severity diagnosis. For TZD- and insulin-medicated patients, there are considerable differences in the 1800–900 cm^−1^ spectral region. The increasing absorption band at 1743 cm^−1^, corresponding to *v*CHO aldehyde groups, strongly supports the suggestion that lipid hydroperoxidation most likely occurred due to oxidative stress from COVID-19 in TZD- and insulin-medicated patients. These bands were less pronounced in MET-medicated patients (Figure 1). Overall, the observed changes in FTIR “diagnostic bands” and the developed mathematical simulation model reflect the influence of antidiabetic medication on the SARS-CoV-2 binding affinity to diabetic patients.

## 4. Materials and Methods

### 4.1. Mathematical Model

The traditional Susceptible–Infected–Recovered (SIR) model, modified to include fractional-order calculus, served as the foundation of our new mathematical model [12,21]. Numerical computations were performed using the Garrappa Predictor–Corrector Method (PECE), which was developed using an algorithm based on the fractional form of the Adams–Moulton method for fractional differential equations (FDEs) [22]. The parameters β = 0.27 and θ = 0.31 were used to estimate our requirements and numerically approach the issue. The mathematical computation data were analyzed with the MATLAB (code R2024b) software. The patients in the model are divided into three groups based on antidiabetic medications: thiazolidinedione (TZD), insulin, and metformin (MET). The number of infected and recovered T2D patients and their antidiabetic medications, used to estimate the impact of COVID-19, were provided by the Greek National Public Health Organization (GNPHO) and the collected clinical data [25,26]. Table 1 shows the major characteristics of the patients [26].

### 4.2. FTIR Spectroscopy

FTIR spectroscopy is a non-destructive physicochemical method that does not require any special sample preparation. For the present study, 6 sections were used, each 10 μm thick and 5 mm long calcified coronary artery sections from 6 T2D patients, who underwent endarterectomy during coronary artery bypass grafting (CABG). The samples were fixed in formalin solution and used without further special preparation, as described in previous publications [1,39]. The samples were taken after surgery according to the Declaration of Helsinki, the Greek ethical rules for ex vivo clinical research studies, and the permission of the Hospital (19912/21-04-2020).

## 5. Conclusions

In the present study, the developed mathematical simulation models and FTIR spectroscopic experimental findings provide the association between pathophysiological properties of glucose-lowering drugs with respect to COVID-19 protection. The intensity decrease in the *v*NH stretching vibration band near 3300 cm^−1^, in turn, reduces the α-helix native protein caused by the disease. The new “diagnostic band” near 1775 cm^−1^, attributed to IgG antibodies, could be used to diagnose those infected by COVID-19. Metformin seems to act both directly through hydrogen bonds with the SARS-CoV-2 spike protein and indirectly by regulating the AMP-protein kinases. Thiazolidinediones act on the P450 enzyme by transferring electrons to it with a mechanism that, under oxidative stress, upregulates its redox potential, leading to altered inflammation induced by viruses. Finally, the application of physicochemical FTIR spectroscopy in biology and medicine provides “diagnostic bands” that are important for COVID-19 diagnosis. The most important absorption bands are found at 1775 cm^−1^ and 1743 cm^−1^, which are assigned to IgG glycosylated carbonyl and CHO aldehyde groups, respectively, and could be used as “diagnostic bands” for COVID-19 progression. Both the proposed mathematical model and FTIR spectroscopy data aligned with clinical and epidemiological data.

According to the mathematical model and FTIR spectral data, we suggest that the potential development of a COVID-19 antivirus drug should contain NH binding sites.

## Figures and Tables

**Table 1:** Characteristics of the patients used [26].

Total	Hospitalized	Deaths
339	276	63
Metformin	144	21
Insulin	72	14
TZD	60	25

## Data Availability

No new data were created or analyzed in this study. Data sharing is not applicable to this article.

## Abbreviations

The following abbreviations are used in the manuscript:

ACE2	Angiotensin-Converting Enzyme 2
AGEs	Advanced Glycation End Products
Arg	Arginine
ATPase	Adenosine Triphosphate kinase (enzyme)
FOC	Fractional-Order Calculus
FTIR	Fourier-Transform Infrared
GNPHO	Greek National Public Health Organization
H-bond	Hydrogen Bond
IgG	Immunoglobulin
MET	Metformin
NMR	Nuclear Magnetic Resonance
ODE	Ordinary Differential Equations
PECE	Predictor Corrector
SIR	Susceptible–Infected–Recovered
Tyr	Tyrosine
TZD	Thiazolidinedione
